# Supplementary material for: Factors shaping subjective financial well-being in emerging adults: A comparative study of Italy and Germany
Source: PLoS One. 2025 Apr 21;20(4):e0320725. doi: 10.1371/journal.pone.0320725 (PMC12011218; doi:10.1371/journal.pone.0320725)
Supplement: S1 File — This file contains the Trust in Financial Professionals Scale, developed specifically for this study to assess individuals’ trust in financial professionals. [file pone.0320725.s001.pdf]

Appendix S1

Trust in Financial Professionals scale

“ Evaluate how much each affirmation describes you, from 1 (it does not describe me at all) to 5 (it totally describes me) with an intermediate point (3 = it describes me moderately). “

1.It does not describe me at all		3.It moderately describes me		5.It totally describes me
○	○	○	○	○

1. I trust when a banking professional, proposes me a financial product

2. I have heard about many bank failures, I prefer avoiding start investing my money in bank proposal

3. I trust a financial professional when I know him, and he shows empathy to me

4. I trust a financial professional when they show competence and they explain to me everything

5. I trust a financial professional who uses adequate language that I can understand

6. I do not trust a financial professional who tries to sell me something

7. I do not trust starting a private retirement fund because there are always too many constraints/loopholes

8. Financial professionals always do their best, not the customer’s best

9. I do not trust starting a private retirement fund because laws could change in a long time, and a private retirement fund could turn out to be a disadvantage

10. I don’t trust insurance companies and financial companies in the country where I live

because they take advantage of bureaucracy to earn at our expenses
