# Supplementary material for: Factors shaping subjective financial well-being in emerging adults: A comparative study of Italy and Germany
Source: PLoS One. 2025 Apr 21;20(4):e0320725. doi: 10.1371/journal.pone.0320725 (PMC12011218; doi:10.1371/journal.pone.0320725)
Supplement: Attachment — Submitted filename: Miccolietal_Rebuttal letter.pdf [file pone.0320725.s004.pdf]

Tuesday, 28 January 2025

Authors‘ Answer to the Reviewer and Academic Editor’s Comments

Dear Dr. José Manuel Santos Jaén, and Reviewer,

Thank you very much for your thoughtful feedback. Your review has been valuable in identifying areas for improvement, and we appreciate the effort you and the reviewer have invested in providing constructive insights.

We agree that addressing the specific points raised will enhance the clarity, rigor, and applicability of our findings. We are committed to making revisions that will strengthen the overall contribution of our work to the field of subjective financial well-being in emerging adults.

In the revised manuscript, we have now refined the argument by refining the connection between the data and the conclusion based on how the study was conducted. Specifically, we have clarified our discussion to ensure that the methodological underpinnings and their implications are transparent and well-articulated, including a section on “Limitations and Future Research” and refining the “Theoretical and practical implications” section. Additionally, we further clarify that the data are fully available on OSF (https://osf.io/hmqfe/), include a visualization to enhance readers’ understanding of key concepts, make clear the rigor of the statistical analyses, and improve the flow of the “Conclusions” section. In the marked-up copy of the manuscript ('Revised Manuscript with Track Changes'), all the changes are marked up in dark blue.

Below, we report the itemized summaries provided under the “Review Comments to the Author” section and our responses (in bold) to your and the reviewer’s comments.

We hope that these revisions address the concerns raised and hope they meet your expectations. We are available for any further clarifications.

Kind regards,

Maria Rosa Miccoli, Yury Shevchenko, Paola Iannello and Ulf-Dietrich Reips

---

Answers to the reviewer:

- In the Abstract, the expression "F(3, 371) = 6.53, p = .002" is unnecessary and can be omitted.

Done.

- In the section Theoretical Analysis and Hypothesis Development, adding a visual representation or diagram would help readers understand the concepts more effectively.

On page 8, we have now included a visual representation [Fig 1] of the factors hypothesized to be related to subjective financial well-being. The figure has been uploaded to the submission portal.

- In the Analysis section, when introducing multiple regression analysis, it would be beneficial to provide the exact regression equation used in the study.

Done [p. 16]. We constructed two multiple linear regression models. Model 1 includes variables identified in the framework proposed by Kempson and colleagues. Model 2 builds upon Model 1 by incorporating additional variables, specifically trust, and maximization, to examine their effects on subjective financial well-being. We tested the fundamental assumptions of multiple regression. Reliability measures for the survey scales were included to demonstrate the soundness of the instruments used.
The data and scripts used for the analyses are publicly available on the Open Science Framework project (https://osf.io/hmqfe/), and this has now been clarified in the manuscript.

- The Conclusion section should include a discussion of the study's limitations.

Thank you for your valuable suggestion. We now included a "Limitations and future research" paragraph [p. 23-25] in the discussion section, where we specify the limitations of our study to facilitate the interpretation of our results. We have also briefly referenced the key limitations in the Conclusion [p. 27] to further align with your suggestion.

- In the Conclusion section, the suggestions for "further research" should be more specific and detailed.

Done. We further increased the specificity of the suggestions for future research, and we moved such suggestions from the "Theoretical and Practical implications" to the new paragraph "Limitations and future research" [p. 23-25].

- It would be better to switch the order of the Theoretical and Practical Implications section and the Conclusions section for improved flow and readability.

Thank you for your suggestion. We have switched the order of the sections to improve flow and readability, as recommended. In alignment with guidelines for scientific writing, the "Theoretical and Practical Implications" section has now been integrated with the Discussion, where it logically complements the interpretation of findings [p.25-26]. Additionally, we have further refined the Conclusions section to summarize the study's key contributions and takeaways.
